# Combination stem cell therapy for heart failure

**DOI:** 10.1186/1755-7682-3-5

**Published:** 2010-04-14

**Authors:** Thomas E Ichim, Fabio Solano, Fabian Lara, Jorge Paz Rodriguez, Octav Cristea, Boris Minev, Famela Ramos, Erik J Woods, Michael P Murphy, Doru T Alexandrescu, Amit N Patel, Neil H Riordan

**Affiliations:** 1 Medistem Inc, San Diego, USA; 2 Cell Medicine Institutes, San Jose, Costa Rica; 3 Cell Medicine Institutes, Panama City, Panama; 4 University of Guelph, Guelph, Canada; 5 Moores Cancer Center, UCSD, San Diego, USA; 6 General BioTechnology, LLC, Indianapolis, USA; 7 Division of Vascular Surgery, Indiana University School of Medicine, Indiana, USA; 8 Georgetown Dermatology, Washington DC, USA; 9 Dept of Cardiothoracic Surgery, University of Utah, Salt Lake City, USA

## Abstract

Patients with congestive heart failure (CHF) that are not eligible for transplantation have limited therapeutic options. Stem cell therapy such as autologous bone marrow, mobilized peripheral blood, or purified cells thereof has been used clinically since 2001. To date over 1000 patients have received cellular therapy as part of randomized trials, with the general consensus being that a moderate but statistically significant benefit occurs. Therefore, one of the important next steps in the field is optimization. In this paper we discuss three ways to approach this issue: a) increasing stem cell migration to the heart; b) augmenting stem cell activity; and c) combining existing stem cell therapies to recapitulate a "therapeutic niche". We conclude by describing a case report of a heart failure patient treated with a combination stem cell protocol in an attempt to augment beneficial aspects of cord blood CD34 cells and mesenchymal-like stem cells.

## Introduction

For the approximately 5 million Americans with heart failure, of which only a small proportion are eligible for transplantation, regenerative medicine is the only therapeutic hope. CHF is caused by many factors, such as poor perfusion due to atherosclerotic disease, a previous heart attack, a congenital defect, or previous viral infection, but the end result is usually similar: a self perpetuating cycle of cardiomyocyte death, inflammatory mediator release, myocardial compensatory hypertrophy, and additional cardiomyocyte death, culminating in a deterioration of ejection fraction. Numerous common themes are associated with the progression to heart failure. We will discuss below how stem cell therapy may act on these factors in a therapeutic sense.

## Inhibition of Inflammatory Cascade by Mesenchymal Stem Cells

Ongoing inflammation is part of the cascade leading to heart failure. Acute inflammation occurs during infarction as a result of tissue damage, however, chronic inflammatory markers are present in both post-infarct patients, as well as ischemic heart failure patients, and patients with congenital defects. In general, a positive correlation between advanced heart failure and levels of the inflammatory marker C-reactive protein (CRP) has been reported [1,2]. While CRP elevation is conventionally seen as a marker of ongoing inflammation, produced by the liver in response to cytokines such as IL-1, IL-6, and TNF-alpha [3], it also plays an active role in cardiac deterioration through induction of endothelial dysfunction [4,5], as well as exacerbation of inflammatory processes through activation of complement [6,7]. In addition to CRP, elevated levels of inflammatory cytokines are also noted in CHF patients [8]. Inflammatory mediators are produced not only as a result of cardiomyocyte ischemia, but also stretch injury as a result of hypertrophic accommodation [9,10] and systemic activation of immune cells including T cells [11] and monocytes [12]. Functionally, inflammatory mediators induce direct apoptosis of cardiomyocytes. For example TNF-alpha is known to induce reduction of bcl-2 gene expression and activate caspase-dependent apoptosis in cardiac cells at physiological concentrations [13]. Reduction of TNF-alpha activity using soluble receptors has demonstrated beneficial effects in animal models of heart failure [14].

The importance of inflammatory stimuli in heart failure can be seen in animal models in which activators of inflammatory agents, such as toll-like receptors (TLRs) are knocked-out. Generally, TLRs particularly TLR 2 and 4, recognize endogenous "danger signals" associated with damaged tissue such as extracellular matrix degradation products [15,16], and heat shock proteins [17]. Doxorubicin induced heart failure is substantially attenuated in animals lacking TLR-2 [18] or TLR-4 [19]. TLR-2 knockout mice have a substantially better clinical outcome after experimental infarction, including reduction in remodeling, wall thinning, and preservation of LVEF as compared to wild-type controls [20]. Clinically, expression of TLR-4 is associated with poor prognosis in post-infarct patients [21]. Thus it appears that inflammation is associated with progression of heart failure. Supporting an "innate" immune component to heart failure are studies by Linden's group demonstrating that NKT cells play a fundamental role in reperfusion injury, and that modulation of these cells with adenosine receptor agonists results in cardioprotection [22,23].

Mesenchymal stem cells (MSC) were originally identified as "stromal cells", believed to play a role in shaping the bone marrow microenvironment where hematopoiesis occurs [24]. More recently, MSC-like populations have been isolated from a diverse range of sources such as adipose [25], heart [26], Wharton's Jelly [27], dental pulp [28], peripheral blood [29], cord blood [30], and more recently menstrual blood [31-33]. In addition to potent regenerative activities of MSC, which we will describe below, MSC have potent anti-inflammatory activities which appear to be present regardless of tissue of origin [34,35]. Mechanistically, MSC appear to suppress inflammation through secretion of anti-inflammatory cytokines such as IL-10 [36], TGF-beta [37], LIF [38], soluble HLA-G [39] and IL-1 receptor antagonist [40], expression of immune regulatory enzyme such as cycloxygenase [41] and indolamine 2,3 deoxygenase [42], and ability to induce generation of anti-inflammatory T regulatory cells [43]. The in vivo anti-inflammatory effects of MSC may be witnessed by success in treating animal models of immune mediate/inflammatory pathologies such as multiple sclerosis [44], colitis [45], graft versus host [46], rheumatoid arthritis [47], and ischemia/reperfusion injury [48]. In heart failure, administration of MSC post infarct has been demonstrated to decrease production of TNF-alpha and IL-6, but upregulate generation of the anti-inflammatory cytokine IL-10, which correlated with therapeutic benefit [49]. Clinically, MSC have demonstrated repeatedly potent therapeutic activity at suppressing graft versus host (GVHD) [50-55]. Thus one angle in which stem cell therapy may be useful for heart failure is by suppressing ongoing self-perpetuating inflammatory cascade.

## Inhibition of Death/Repair

Cardiomyocyte death, either by apoptosis [56], or other types of death such as autophagy and programmed necrosis is part of the self-perpetuating cascade leading to heart failure [57,58]. Thus the manipulation of these death pathways, and upregulation of endogenous repair mechanisms in the heart could be a possible method of decreasing the progression to heart failure. For example, suppression of intracellular apoptotic pathways, as performed by transgenic expression of a dominant negative form of Mammalian sterile 20-like kinase-1 (Mst1), has been shown to inhibit post infarct remodeling [59]. Similar protective effects can be attained by transfection of anti-apoptotic genes such as IAP-2 [60], or growth factors that inhibit apoptosis [61]. ACE inhibitors have been postulate to have some beneficial effects through inhibition of cardiomyocyte apoptosis [62]. Thus one method of addressing the progression to heart failure would be identification of methods to prevent ongoing cell death.

Cell death in the heart causes some level of replacement by resident cardiac stem cells (CSC). These cells are relatively rare and are believed to respond to signals associated with damage to the myocardium. Fransioli et al generated a transgenic mouse expressing GFP under control of the c-kit promoter. Subsequent to infarct, increased proliferation of c-kit positive cells was seen in the myocardium [63]. In humans Urbanek et al examined 20 human hearts from patients who died after acute infarct, 20 hearts with chronic infarct that were transplanted, and 12 control hearts. A population of cells expressing c-kit, MDR1 and Sca-1 were seen to enter cell cycle from a basal rate of 1.5% cycling cells in controls, to 28% and 14% in acute and chronic infarcts, respectively. The cells expressing the phenotype were demonstrated to be capable of differentiating into myocyte, smooth muscle, and endothelial cell lineages [64]. Isolated CSC have been successfully expanded ex vivo and administered via the intracoronary route in rats post-infarct. Successful transmigration of the CSC across the endothelium and active regeneration of myocardium was demonstrated [65]. Thus it appears that a functional population of stem cells exists in the heart that to some extent can cause regeneration post injury.

Both HSC and MSC are capable of secreting factors that on the one hand inhibit apoptosis [66-68] and on the other hand stimulate activation of CSC [69]. For example, it was demonstrated that administration of non-fractionated bone marrow cells containing both cell populations protects against apoptosis in a doxorubin induced cardiomyopathy model [70]. Furthermore bone marrow cells are known to produce HGF [71] and IGF-1 [72], cytokines that are anti-apoptotic and activate endogenous cardiomyocyte stem cells [69]. Interestingly, production of these factors is upregulated in response to inflammatory mediators associated with heart failure such as TNF-alpha [68]. Therefore it may be possible that MSC not only migrate to injured tissue but can also "sense" inflammatory stimuli such as TNF-alpha and actually try to grade the level of their therapeutic response according to the level of damage sensed.

Another means by which stem cells may repair the heart is through actually differentiating into new heart muscle. Reports exist of both hematopoietic [73] and mesenchymal stem cells [74,75] differentiating into cardiac-like cells, although this is controversial and some groups have reported this to be a product of cell fusion [76]. Additionally, stem cells promote angiogenesis, thus providing nutrients to ischemic areas and potentially allowing regeneration [77,78].

## Currently Stem Cell Therapy Helps Heart Patients: Just Not That Well

Cardiac stem cell therapy was first described 2001 when Strauer et al reported a case in which a 46 year old patient received autologous bone marrow mononuclear cells by a percutaneous transluminal catheter placed in the infarct-related artery. 10 weeks after administration the transmural infarct area had been reduced from 24.6% to 15.7% of left ventricular circumference, while ejection fraction, cardiac index and stroke volume had increased by 20-30% [79]. A subsequent paper in the same year reported administration of similar cells in 5 patients with advanced ischemia undergoing coronary artery bypass grafting. Cells were administered intramuscularly into areas deemed ungraftable and perfusion was assessed by imaging. Specific improvement in areas injected was documented in 3 of the 5 patients. No ectopic growths or adverse effects were reported at 1 year follow-up [80]. Since these pioneering studies, cardiac stem cell therapy has been used by numerous groups for numerous conditions causing heart failure. These can be broken down into: a) inhibiting post acute myocardial infarction remodeling; b) stimulation of regeneration in chronically injured hearts and c) induction of angiogenesis in coronary artery disease. The methods of administering stem cells have included the intracoronary, epicardial, and intravenous routes. Stem cells used to date are bone marrow mononuclear cells, mobilized peripheral blood stem cells, purified CD34 or CD133 cells, autologous mesenchymal stem cells, and allogeneic bone marrow and placental mesenchymal stem cells.

To avoid detailed examination, we will discuss several meta-analysis of ongoing clinical trials performed. Abdel-Latif et al described 999 patients enrolled 18 independent controlled cardiac trials in which patients were treatment with either unseparated bone marrow cells, bone marrow mesenchymal, or mobilized peripheral blood [81]. They found that in comparison to controls, there was a statistically significant improvement in ejection fraction, reduction in infarct size and left ventricular end-systolic volume. Importantly, no safety issues or serious treatment associated adverse events were noted. In another such comprehensive review, Martin-Rendon et al focused on bone marrow therapy for post acute infarction trials. Of 13 randomized studies conducted, encompassing 811 participants, the authors of the review stated that more trials are needed to establish efficacy in terms of clinical endpoints such as death. However that authors of the review did observe a consistent improvement in LVEF, as well as trends for decrease in left ventricular end systolic and end diastolic volumes, and infarct size [82]. Two other meta-analysis of randomized trials in the area of bone marrow stem cell infusions also supported the conclusion of safety and mild but statistically significant improvement in LVEF [83,84]. These data suggest that stem cell therapy, both hematopoietic and mesenchymal have clinical effects in various types of heart failure. Theoretically the leap between these clinical trials and widespread implementation is more of a business question than a medical question. In order to postulate on the future of cardiac stem cell therapy, we will discuss several possible means of optimizing existing work.

## How to Increase Stem Cell Efficacy?

Attempts at increasing efficacy of stem cells for cardiac indications have taken several avenues of investigation: increasing trafficking efficacy; enhancing plasticity of administered cells; and increasing growth factor production. Endowment of these features as been performed by gene transfection or modification of culture conditions such as exposure to cytokines or hypoxia. Another interesting approach is addition of chemotactic agents to the area of tissue injury to enhance trafficking. These approaches will be discussed below.

## Making Stem Cells Home Better

Mesenchymal stem cells are known to migrate to injured tissue and hypoxic tissue through expression of receptors such as CD44 [85-87] and CXCR-4 [88], respectively. One method of increase efficacy of these cells is to increase their ability to traffic to where they are needed. This has been performed using various approaches. Cheng et al used retroviral transfection to overexpress CXCR-4 on rat bone marrow derived MSC. These cells were functionally competent as judged by similar growth profiles and differentiation ability when compared to control transfected MSC. Intravenous administration of the modified cells in a rat model of myocardial infarction led to a significant improvement in migration to the area of infarct, and LVEF, as well as decreased wall thinning and fibrosis when compared to animals receiving control MSC [89]. Although many fears exist surrounding genetically modified cells, current advances in delivery vectors have for increased safety features which may allow such modified MSC to become a clinical reality [90]. An alternative and perhaps easier way of inducing MSC to expression CXCR-4 is simply "pulsing" them with a brief period of hypoxia [91], or exposure to cytokines such as SCF, IL-6, Flt-3 ligand, HGF and IL-3 [92].

Instead of increasing affinity of the stem cells to the chemoattractant, the other way to achieve the same result is to increase the concentration of the chemoattractant. One way is to provide an exogenous depot of angiogenic cytokines in proximity to the area where stem cell migration is desired. Tang et al administered a SDF-1 expressing plasmid into the ischemic border zone 2 weeks after induction of infarct in BALB/c mice. To determine whether the expressed chemoattractant actually caused stem cell homing, syngeneic labeled bone marrow cells were intravenously injected 3 days after SDF-1 plasmid administration. A significantly increased number of labeled cells was observed in the group receiving the plasmid, in the area whether the plasmid was injected [93]. These data suggest that it is feasible to reproduce mobilization induced by infarcts through the administration of homologous cytokines. However, the authors did not describe therapeutic benefit. In another experiment, a more clinically-translatable approach was taken. Fibrin glue, fibrinogen and thrombin mixed at the point of care, is used in surgery to control bleeding [94]. Zhang et al used pegylation technology to covalently bind recombinant SDF-1 to fibrinogen and demonstrated that subsequent to mixing with thrombin, the resultant "patch" could serve as a means of controlled release of SDF-1. The patch was placed on the infarct area of the left ventricle of mice after ligation of the left anterior descending coronary artery. In comparison to control mice receiving a fibrin patch lacking SDF-1, an increase in cells with a stem cell antigen and c-kit positive phenotype was observed in the experimental group. Additionally, at completion of experiment, an increased LVEF was observed in the treatment mice [95]. Since endogenous cardiac stem cells also express a similar phenotype [96], and cell labeling was not performed, it is difficult to determine whether the therapeutic effect was mediated by mobilization of bone marrow progenitors or cardiac resident stem cells. One interesting way of enhancing activity of such a localization of chemoattractants is to concurrently administer exogenous stem cells, or to mobilize endogenous bone marrow stem cells. In fact, the latter was performed in a study where fibroblasts expressing SDF-1 were injected into the hindlimbs of mice after femoral ligation. A synergistic induction of angiogenesis was detected when endogenous bone marrow derived stem cells were mobilized with G-CSF [97]. Other clinically used methods may be implemented to enhance stem cell trafficking. For example, erythropoietin (EPO), in addition to its well-known anti-apoptotic effects on cardiomyocytes [98], has actually been shown to stimulate responsiveness of bone marrow derived stem cells to SDF-1 when administered in vivo [99]. A recent paper described the nutraceutical Stem-Kine as the first food supplement capable of augmenting endogenous circulating stem cells, this approach may spare patients potential adverse effects associated with cytokine mobilization [100]. The procedure of transmyocardial revascularization has been demonstrated to synergize with endothelial progenitor cells for augmentation of neoangiogenesis [101], it remains an open question whether other stem cell therapies may synergize with this therapy. Combination therapies of this sort will be interesting to evaluate clinically, especially when the various components are already approved.

## Revitalize Stem Cells

Once we can make sure that stem cells arrive to the site where they are needed to stimulate regeneration, how do we know that they can do this effectively? For example, we do know that in general, stem cell activity diminishes with age [102], and specifically, in patients with cardiovascular risk factors stem cell activity is additionally suppressed as compared to healthy age-matched controls [103]. There are several issues that must be taken into consideration. Perhaps, most importantly, is how do the stem cells mediate their therapeutic effects? On the one hand, people will state that adult stem cells, such as hematopoietic [104] and even in some cases mesenchymal stem cells [105], do not differentiate into functional cardiomyocytes, so therefore therapy with these cells is a futile endeavor. As we discussed above, efficacy of cardiac stem cell therapy does not rely on cell replacement but could be, and most likely is, mediated by trophic, angiogenic, anti-inflammatory and anti-apoptotic effects. Regardless of this, the concept of "revitalizing" an adult stem cell so to be able to actually replace cardiac cells is very exciting.

One method of such "revitalization" is involves making the stem cells take a more primitive, embryonic stem cell-like phenotype. It is known that the more differentiated cells become, the less plasticity they have, and the more restricted epigenetically, they become. Perhaps this was associated with the reason why DNA methyltransferase inhibitors such as 5-azacytidine were initially added to stem cells before implantation into infracted hearts [106,107]. Other agents that act epigenetically, such as the histone deacetylase inhibitor valproic acid have been demonstrated to enhance hematopoietic stem cell self renewal capacity in vitro [108,109], and have a positive effect on post infarct remodeling in vivo, although it is not clear whether stem cell activation is implicated [110]. Instead of using agents such as these that upregulate factors associated with pluripotency such as Nanog [111], an alternative approach is to simply transfect the cells with such genes. For example Go et al transfected bone marrow derived MSC with Nanog and reported superior expansion potential and ability to differentiate as compared to control transfected cells [112]. Transfection of such "retrodifferentiation" genes is particularly exciting in light of the recent discovery that fibroblasts can be induced to pluripotency through introduction of the pluripotency genes Oct3/4, Sox2, c-Myc, and Klf4 in mice [113] and humans [114]. These "inducible pluripotent stem cells" (iPS) appear to be functional, not only by gene transcription profile, but also ability to reconstitute animals hematopoietically [115]. Theoretically, it would make sense that retrodifferentiation of an adult stem cell into an iPS would be easier than a skin fibroblast. Indeed, Kim et al demonstrated that in order to derived iPS cells from neural stem cells, only the factors Oct-4 and klf-4 or c-Myc are needed [116]. Furthermore, newer transfection methods of generating iPS through non-retroviral means have been reported, giving the possibility of generating clinically applicable therapies from these cells [117]. Unfortunately, carcinogenesis associated with the viral vectors is not the main limitation. It is known in general that ES cells are carcinogenic [118]. Additionally, the very transcriptional profile associated with cancer stem cells appears to be related to that of pluripotent cells, regardless if they are generated by iPS or from ES cells [119].

Thus one way of increasing potency of MSC-based therapy is through induction of such a "rejuvenation" unfortunately, too much rejuvenation leads to the possibility of carcinogenesis, and additionally may have implications on ability of the cells to evade immune responsiveness and/or migration to the area of injury. For example, it is known that embryonic stem cells are hypoimmunogenic, as seen by weak ability to stimulate allogeneic lymphocyte proliferation [120]. However it remains an open question whether ES cells can actively suppress ongoing immune responses as is the case with MSC both in animal models [45] and clinically [121,122]. In terms of migratory ability, it is known that functionally various adult stem cells play a protective role in the physiological response to injury. Although the effects in clinical situations are minor, there is suggestive evidence, for example in stroke patients that a correlation between endogenous stem cell mobilization and positive outcome exists [123,124]. While in cardiac infarct cases we do know that mobilization occurs [125], but correlation with infarct recovery have not been made. Regardless, the question of what stage of differentiation the best cell population is for treatment of cardiac indications remains unclear.

## Stem Cell Combinations

Given that we do not know the best stage of differentiation to administer the stem cells, as well as the various drawbacks of transfection and reprogramming approaches, one possible way of advancing efficacy of stem cell therapy would be to combine various stem cell types that we know have trophic activity. One interesting combination would be the use of CD34 cells, which are primarily hematopoietic, but also angiogenic, together with allogeneic mesenchymal stem cells, which have trophic, angiogenic, and potent anti-inflammatory potential. The rationale for combining these two approaches come from several perspectives: a) after tissue injury both mesenchymal [85,126,127] and hematopoietic stem cells [128-130] are mobilized thus potentially both cells may have therapeutic synergistic activity in a physiological sense; b) In vivo MSC provide a microenvironment for CD34 stem cells both embryonically [131], and postnatally [132], in vitro MSC promote expansion of CD34 stem cells [133,134]; and c) animal models suggest synergy of function [135].

We have previously published data from an end-stage patient suffering from dilated cardiomyopathy which underwent a profound improvement in ejection fraction after receiving a combination of cord blood expanded CD34 cells and placental matrix derived mesenchymal stem cells [136]. In the current case report we describe a patient with ischemia cardiomyopathy who received a combination of allogeneic CD34 cells and endometrial regenerative cells (ERC), a MSC-like population which has previously been demonstrated to possess higher growth factor production ability as compared to control MSC cells [31], as well as in immunomodulatory and in vivo angiogenic activity [137]. Furthermore, ERC-like cells have been reported by independent groups to possess an increased propensity towards muscular [138,139] and cardiac differentiation [140], as compared with other stem cell types. Animal safety studies have demonstrated that ERC do not cause tumors in immune competent animals and actively suppress glioma growth in vivo [141]. Feasibility-based clinical investigations have demonstrated ERC do not cause abnormal growths subsequent to intrathecal or intravenous administration [142].

## Case Report

Endometrial Regenerative Cells (ERC) were provided with a certificate of analysis describing purity (> 90% expression of CD90 and CD105, and < 5% expression of CD34 and CD45) and sterility (lack of adventitious contaminants). ERC and cord blood CD34 cells were generated described in our previous publications [136,143].

Patient was born January 7^th^, 1933. At 74 the patient presented with congestive heart failure and an ejection fraction of 25-30%. Risks associated with stem cell therapy and specifically with the combination of two stem cell products, of which neither one has been approved by the FDA, or EMEA for general use were explained to the patient in detail. After signing informed consent and being accepted by an independent oversight committee for the experimental treatment, the patient was administered ERCs. On days 1,2,3,4 and 7 the patient received intravenous administration of 3 million ERC (total number 15 million). CD34 cells were administered as follows: day 1 (4.5 million); day 2 (3 million); day 3, (2.3 × 10(5)); day 4 (1.5 million); and day 7 (1.5 million). Last injection was performed November 19^th^, 2007. Injection was performed by intravenous drip using USP-grade saline and autologous heat inactivated serum (10%). Injection site and general condition of the patient was monitored for 3 hours after the first administration. Before subsequent injections the injection site was examined for swelling or inflammation and the general state of the patient was examined. For all injections no evident reactions were noted.

Echocardiogram performed June 27, 2008, August 11, 2008, and Oct 1^st ^2009 demonstrated that the patient's ejection fraction was approximately 40%. The Minnesota Living with Heart Failure Questionnaire score dropped from 97 pretreatment to a value of 2 onFebruary 2009. Reduction of Pro-BNP was observed after treatment: Pre-treatment levels of Pro-BNP were 1946 on September 5^th^, 2007, 1225 on January 11, 2008, and 788.1 on Sept 28, 2009. The patient has other chemical and metabolic testing to further analyze his heart failure. Complete blood count, serum biochemistry, PSA, CEA, alpha fetoprotein, fecal occult blood test revealed no abnormalities in comparison to reference ranges. This was similar to baseline. Radiological examination of the chest PA and Lateral x-ray did not reveal any abnormalities. Physical examination of the patient, with special emphasis on the injection site revealed no masses, inflammation or abnormalities. This patient continues to do well.

## Conclusion

Adult stem cell therapy for cardiac conditions has reached the point where new directions are needed to optimize effects. Possibilities of next-generation approaches include the use of "in vitro supercharged" cells, combinations of cells and cytokines, and of course combination of cellular therapies. Currently the use of endometrial regenerative cells as a possible substitute for bone marrow mesenchymal stem cells is being evaluated by our group including novel methods to image the cells and the changes in heart function.

## Competing interests

NHR and TEI are shareholders and management of Medistem Inc. FS, FL and JPR were employees of Cell Medicine Institutes Costa Rica, and Panama, respectively at time of manuscript submission.

## Authors' contributions

TEI, FS, FL, JPR, OC, BM, FM, EJW, MPM, DTA, ANP, and NHR performed literature review and wrote the manuscript. FS, FL, and JPR reported on the clinical case. All authors read and approved the final manuscript.

## References

[B1] Sanchez-Lazaro IJ Almenar L Reganon E Vila V Martinez-Dolz L Martinez-Sales V Moro J Aguero J Ortiz-Martinez V Salvador A Inflammatory markers in stable heart failure and their relationship with functional class Int J Cardiol 2007 129 3 388 93 10.1016/j.ijcard.2007.07.138 18022711

[B2] Alonso-Martinez JL Llorente-Diez B Echegaray-Agara M Olaz-Preciado F Urbieta-Echezarreta M Gonzalez-Arencibia C C-reactive protein as a predictor of improvement and readmission in heart failure Eur J Heart Fail 2002 4 331 336 10.1016/S1388-9842(02)00021-1 12034159

[B3] Nakou ES Liberopoulos EN Milionis HJ Elisaf MS The role of C-reactive protein in atherosclerotic cardiovascular disease: an overview Curr Vasc Pharmacol 2008 6 258 270 10.2174/157016108785909733 18855714

[B4] Galarraga B Khan F Kumar P Pullar T Belch JJ C-reactive protein: the underlying cause of microvascular dysfunction in rheumatoid arthritis Rheumatology (Oxford) 2008 47 12 1780 4 10.1093/rheumatology/ken386 18854346

[B5] Nabata A Kuroki M Ueba H Hashimoto S Umemoto T Wada H Yasu T Saito M Momomura S Kawakami M C-reactive protein induces endothelial cell apoptosis and matrix metalloproteinase-9 production in human mononuclear cells: Implications for the destabilization of atherosclerotic plaque Atherosclerosis 2008 196 129 135 10.1016/j.atherosclerosis.2007.03.003 17531242

[B6] Griselli M Herbert J Hutchinson WL Taylor KM Sohail M Krausz T Pepys MB C-reactive protein and complement are important mediators of tissue damage in acute myocardial infarction J Exp Med 1999 190 1733 1740 10.1084/jem.190.12.1733 10601349 PMC2195725

[B7] Pepys MB Hirschfield GM Tennent GA Gallimore JR Kahan MC Bellotti V Hawkins PN Myers RM Smith MD Polara A Targeting C-reactive protein for the treatment of cardiovascular disease Nature 2006 440 1217 1221 10.1038/nature04672 16642000

[B8] Satoh M Minami Y Takahashi Y Nakamura M Immune modulation: role of the inflammatory cytokine cascade in the failing human heart Curr Heart Fail Rep 2008 5 69 74 10.1007/s11897-008-0012-2 18765076

[B9] Yokoyama T Sekiguchi K Tanaka T Tomaru K Arai M Suzuki T Nagai R Angiotensin II and mechanical stretch induce production of tumor necrosis factor in cardiac fibroblasts Am J Physiol 1999 276 H1968 1976 10362677 10.1152/ajpheart.1999.276.6.H1968

[B10] Wang BW Hung HF Chang H Kuan P Shyu KG Mechanical stretch enhances the expression of resistin gene in cultured cardiomyocytes via tumor necrosis factor-alpha Am J Physiol Heart Circ Physiol 2007 293 H2305 2312 10.1152/ajpheart.00361.2007 17573461

[B11] Satoh S Oyama J Suematsu N Kadokami T Shimoyama N Okutsu M Inoue T Sugano M Makino N Increased productivity of tumor necrosis factor-alpha in helper T cells in patients with systolic heart failure Int J Cardiol 2006 111 405 412 10.1016/j.ijcard.2005.08.021 16271779

[B12] Conraads VM Bosmans JM Schuerwegh AJ Goovaerts I De Clerck LS Stevens WJ Bridts CH Vrints CJ Intracellular monocyte cytokine production and CD 14 expression are up-regulated in severe vs mild chronic heart failure J Heart Lung Transplant 2005 24 854 859 10.1016/j.healun.2004.04.017 15982613

[B13] Haudek SB Taffet GE Schneider MD Mann DL TNF provokes cardiomyocyte apoptosis and cardiac remodeling through activation of multiple cell death pathways J Clin Invest 2007 117 2692 2701 10.1172/JCI29134 17694177 PMC1937497

[B14] Kubota T Bounoutas GS Miyagishima M Kadokami T Sanders VJ Bruton C Robbins PD McTiernan CF Feldman AM Soluble tumor necrosis factor receptor abrogates myocardial inflammation but not hypertrophy in cytokine-induced cardiomyopathy Circulation 2000 101 2518 2525 10831527 10.1161/01.cir.101.21.2518

[B15] Scheibner KA Lutz MA Boodoo S Fenton MJ Powell JD Horton MR Hyaluronan fragments act as an endogenous danger signal by engaging TLR2 J Immunol 2006 177 1272 1281 16818787 10.4049/jimmunol.177.2.1272

[B16] Termeer C Benedix F Sleeman J Fieber C Voith U Ahrens T Miyake K Freudenberg M Galanos C Simon JC Oligosaccharides of Hyaluronan activate dendritic cells via toll-like receptor 4 J Exp Med 2002 195 99 111 10.1084/jem.20001858 11781369 PMC2196009

[B17] Asea A Heat shock proteins and toll-like receptors Handb Exp Pharmacol 2008 111 127 full_text 18071657 10.1007/978-3-540-72167-3_6

[B18] Nozaki N Shishido T Takeishi Y Kubota I Modulation of doxorubicin-induced cardiac dysfunction in toll-like receptor-2-knockout mice Circulation 2004 110 2869 2874 10.1161/01.CIR.0000146889.46519.27 15505089

[B19] Riad A Bien S Gratz M Escher F Westermann D Heimesaat MM Bereswill S Krieg T Felix SB Schultheiss HP Toll-like receptor-4 deficiency attenuates doxorubicin-induced cardiomyopathy in mice Eur J Heart Fail 2008 10 233 243 10.1016/j.ejheart.2008.01.004 18321777

[B20] Shishido T Nozaki N Yamaguchi S Shibata Y Nitobe J Miyamoto T Takahashi H Arimoto T Maeda K Yamakawa M Toll-like receptor-2 modulates ventricular remodeling after myocardial infarction Circulation 2003 108 2905 2910 10.1161/01.CIR.0000101921.93016.1C 14656915

[B21] Sheu JJ Chang LT Chiang CH Youssef AA Wu CJ Lee FY Yip HK Prognostic value of activated toll-like receptor-4 in monocytes following acute myocardial infarction Int Heart J 2008 49 1 11 10.1536/ihj.49.1 18360060

[B22] Yang Z Linden J Berr SS Kron IL Beller GA French BA Timing of adenosine 2A receptor stimulation relative to reperfusion has differential effects on infarct size and cardiac function as assessed in mice by MRI Am J Physiol Heart Circ Physiol 2008 295 H2328 2335 10.1152/ajpheart.00091.2008 18849340 PMC2614530

[B23] Yang Z Day YJ Toufektsian MC Xu Y Ramos SI Marshall MA French BA Linden J Myocardial infarct-sparing effect of adenosine A2A receptor activation is due to its action on CD4+ T lymphocytes Circulation 2006 114 2056 2064 10.1161/CIRCULATIONAHA.106.649244 17060376

[B24] Friedenstein AJ Petrakova KV Kurolesova AI Frolova GP Heterotopic of bone marrow. Analysis of precursor cells for osteogenic and hematopoietic tissues Transplantation 1968 6 230 247 10.1097/00007890-196803000-00009 5654088

[B25] Zannettino AC Paton S Arthur A Khor F Itescu S Gimble JM Gronthos S Multipotential human adipose-derived stromal stem cells exhibit a perivascular phenotype in vitro and in vivo J Cell Physiol 2008 214 413 421 10.1002/jcp.21210 17654479

[B26] Hoogduijn MJ Crop MJ Peeters AM Van Osch GJ Balk AH Ijzermans JN Weimar W Baan CC Human heart, spleen, and perirenal fat-derived mesenchymal stem cells have immunomodulatory capacities Stem Cells Dev 2007 16 597 604 10.1089/scd.2006.0110 17784833

[B27] Chao KC Chao KF Fu YS Liu SH Islet-like clusters derived from mesenchymal stem cells in Wharton's Jelly of the human umbilical cord for transplantation to control type 1 diabetes PLoS ONE 2008 3 e1451 10.1371/journal.pone.0001451 18197261 PMC2180192

[B28] Jo YY Lee HJ Kook SY Choung HW Park JY Chung JH Choung YH Kim ES Yang HC Choung PH Isolation and characterization of postnatal stem cells from human dental tissues Tissue Eng 2007 13 767 773 10.1089/ten.2006.0192 17432951

[B29] He Q Wan C Li G Concise review: multipotent mesenchymal stromal cells in blood Stem Cells 2007 25 69 77 10.1634/stemcells.2006-0335 16973831

[B30] Oh W Kim DS Yang YS Lee JK Immunological properties of umbilical cord blood-derived mesenchymal stromal cells Cell Immunol 2008 18495100 10.1016/j.cellimm.2008.04.003

[B31] Meng X Ichim TE Zhong J Rogers A Yin Z Jackson J Wang H Ge W Bogin V Chan KW Endometrial regenerative cells: a novel stem cell population J Transl Med 2007 5 57 10.1186/1479-5876-5-57 18005405 PMC2212625

[B32] Hida N Nishiyama N Miyoshi S Kira S Segawa K Uyama T Mori T Miyado K Ikegami Y Cui C Novel Cardiac Precursor-Like Cells from Human Menstrual Blood-Derived Mesenchymal Cells Stem Cells 2008 26 7 1695 704 10.1634/stemcells.2007-0826 18420831

[B33] Patel AN Park E Kuzman M Benetti F Silva FJ Allickson JG Multipotent menstrual blood stromal stem cells: isolation, characterization, and differentiation Cell Transplant 2008 17 303 311 10.3727/096368908784153922 18522233

[B34] Le Blanc K Ringden O Immunomodulation by mesenchymal stem cells and clinical experience J Intern Med 2007 262 509 525 10.1111/j.1365-2796.2007.01844.x 17949362

[B35] Keyser KA Beagles KE Kiem HP Comparison of mesenchymal stem cells from different tissues to suppress T-cell activation Cell Transplant 2007 16 555 562 17708345 10.3727/000000007783464939

[B36] Nasef A Chapel A Mazurier C Bouchet S Lopez M Mathieu N Sensebe L Zhang Y Gorin NC Thierry D Identification of IL-10 and TGF-beta transcripts involved in the inhibition of T-lymphocyte proliferation during cell contact with human mesenchymal stem cells Gene Expr 2007 13 217 226 10.3727/000000006780666957 17605296 PMC6032462

[B37] Ryan JM Barry F Murphy JM Mahon BP Interferon-gamma does not break, but promotes the immunosuppressive capacity of adult human mesenchymal stem cells Clin Exp Immunol 2007 149 353 363 10.1111/j.1365-2249.2007.03422.x 17521318 PMC1941956

[B38] Nasef A Mazurier C Bouchet S Francois S Chapel A Thierry D Gorin NC Fouillard L Leukemia inhibitory factor: Role in human mesenchymal stem cells mediated immunosuppression Cell Immunol 2008 253 16 22 10.1016/j.cellimm.2008.06.002 18639869

[B39] Selmani Z Naji A Zidi I Favier B Gaiffe E Obert L Borg C Saas P Tiberghien P Rouas-Freiss N Human leukocyte antigen-G5 secretion by human mesenchymal stem cells is required to suppress T lymphocyte and natural killer function and to induce CD4+CD25highFOXP3+ regulatory T cells Stem Cells 2008 26 212 222 10.1634/stemcells.2007-0554 17932417

[B40] Ortiz LA Dutreil M Fattman C Pandey AC Torres G Go K Phinney DG Interleukin 1 receptor antagonist mediates the antiinflammatory and antifibrotic effect of mesenchymal stem cells during lung injury Proc Natl Acad Sci USA 2007 104 11002 11007 10.1073/pnas.0704421104 17569781 PMC1891813

[B41] English K Barry FP Field-Corbett CP Mahon BP IFN-gamma and TNF-alpha differentially regulate immunomodulation by murine mesenchymal stem cells Immunol Lett 2007 110 91 100 10.1016/j.imlet.2007.04.001 17507101

[B42] Jones BJ Brooke G Atkinson K McTaggart SJ Immunosuppression by placental indoleamine 2,3-dioxygenase: a role for mesenchymal stem cells Placenta 2007 28 1174 1181 10.1016/j.placenta.2007.07.001 17714779

[B43] Casiraghi F Azzollini N Cassis P Imberti B Morigi M Cugini D Cavinato RA Todeschini M Solini S Sonzogni A Pretransplant infusion of mesenchymal stem cells prolongs the survival of a semiallogeneic heart transplant through the generation of regulatory T cells J Immunol 2008 181 3933 3946 18768848 10.4049/jimmunol.181.6.3933

[B44] Kassis I Grigoriadis N Gowda-Kurkalli B Mizrachi-Kol R Ben-Hur T Slavin S Abramsky O Karussis D Neuroprotection and immunomodulation with mesenchymal stem cells in chronic experimental autoimmune encephalomyelitis Arch Neurol 2008 65 753 761 10.1001/archneur.65.6.753 18541795

[B45] Parekkadan B Tilles AW Yarmush ML Bone marrow-derived mesenchymal stem cells ameliorate autoimmune enteropathy independently of regulatory T cells Stem Cells 2008 26 1913 1919 10.1634/stemcells.2007-0790 18420833

[B46] Li H Guo Z Jiang X Zhu H Li X Mao N Mesenchymal Stem Cells Alter Migratory Property of T and Dendritic Cells to Delay the Development of Murine Lethal Acute Graft-Versus-Host Disease Stem Cells 2008 26 10 2531 41 10.1634/stemcells.2008-0146 18635870

[B47] Augello A Tasso R Negrini SM Cancedda R Pennesi G Cell therapy using allogeneic bone marrow mesenchymal stem cells prevents tissue damage in collagen-induced arthritis Arthritis Rheum 2007 56 1175 1186 10.1002/art.22511 17393437

[B48] Semedo P Wang PM Andreucci TH Cenedeze MA Teixeira VP Reis MA Pacheco-Silva A Camara NO Mesenchymal stem cells ameliorate tissue damages triggered by renal ischemia and reperfusion injury Transplant Proc 2007 39 421 423 10.1016/j.transproceed.2007.01.036 17362746

[B49] Du YY Zhou SH Zhou T Su H Pan HW Du WH Liu B Liu QM Immuno-inflammatory regulation effect of mesenchymal stem cell transplantation in a rat model of myocardial infarction Cytotherapy 2008 10 469 478 10.1080/14653240802129893 18608353

[B50] Le Blanc K Frassoni F Ball L Locatelli F Roelofs H Lewis I Lanino E Sundberg B Bernardo ME Remberger M Mesenchymal stem cells for treatment of steroid-resistant, severe, acute graft-versus-host disease: a phase II study Lancet 2008 371 1579 1586 10.1016/S0140-6736(08)60690-X 18468541

[B51] Ning H Yang F Jiang M Hu L Feng K Zhang J Yu Z Li B Xu C Li Y The correlation between cotransplantation of mesenchymal stem cells and higher recurrence rate in hematologic malignancy patients: outcome of a pilot clinical study Leukemia 2008 22 593 599 10.1038/sj.leu.2405090 18185520

[B52] Ball L Bredius R Lankester A Schweizer J Heuvel-Eibrink M van den Escher H Fibbe W Egeler M Third party mesenchymal stromal cell infusions fail to induce tissue repair despite successful control of severe grade IV acute graft-versus-host disease in a child with juvenile myelo-monocytic leukemia Leukemia 2008 22 1256 1257 10.1038/sj.leu.2405013 17972946

[B53] Ringden O Uzunel M Rasmusson I Remberger M Sundberg B Lonnies H Marschall HU Dlugosz A Szakos A Hassan Z Mesenchymal stem cells for treatment of therapy-resistant graft-versus-host disease Transplantation 2006 81 1390 1397 10.1097/01.tp.0000214462.63943.14 16732175

[B54] Le Blanc K Rasmusson I Sundberg B Gotherstrom C Hassan M Uzunel M Ringden O Treatment of severe acute graft-versus-host disease with third party haploidentical mesenchymal stem cells Lancet 2004 363 1439 1441 10.1016/S0140-6736(04)16104-7 15121408

[B55] Muller I Kordowich S Holzwarth C Isensee G Lang P Neunhoeffer F Dominici M Greil J Handgretinger R Application of multipotent mesenchymal stromal cells in pediatric patients following allogeneic stem cell transplantation Blood Cells Mol Dis 2008 40 25 32 10.1016/j.bcmd.2007.06.021 17869550

[B56] Narula J Haider N Virmani R DiSalvo TG Kolodgie FD Hajjar RJ Schmidt U Semigran MJ Dec GW Khaw BA Apoptosis in myocytes in end-stage heart failure N Engl J Med 1996 335 1182 1189 10.1056/NEJM199610173351603 8815940

[B57] Dorn GW Apoptotic and non-apoptotic programmed cardiomyocyte death in ventricular remodelling Cardiovasc Res 2008 81 3 465 73 10.1093/cvr/cvn243 18779231 PMC2721651

[B58] Rodriguez M Lucchesi BR Schaper J Apoptosis in myocardial infarction Ann Med 2002 34 470 479 10.1080/078538902321012414 12523502

[B59] Odashima M Usui S Takagi H Hong C Liu J Yokota M Sadoshima J Inhibition of endogenous Mst1 prevents apoptosis and cardiac dysfunction without affecting cardiac hypertrophy after myocardial infarction Circ Res 2007 100 1344 1352 10.1161/01.RES.0000265846.23485.7a 17395874

[B60] Chua CC Gao J Ho YS Xiong Y Xu X Chen Z Hamdy RC Chua BH Overexpression of IAP-2 attenuates apoptosis and protects against myocardial ischemia/reperfusion injury in transgenic mice Biochim Biophys Acta 2007 1773 577 583 10.1016/j.bbamcr.2007.01.007 17321613 PMC2709410

[B61] Jayasankar V Woo YJ Bish LT Pirolli TJ Chatterjee S Berry MF Burdick J Gardner TJ Sweeney HL Gene transfer of hepatocyte growth factor attenuates postinfarction heart failure Circulation 2003 108 Suppl 1 II230 236 12970238 10.1161/01.cir.0000087444.53354.66

[B62] Filippatos G Uhal BD Blockade of apoptosis by ACE inhibitors and angiotensin receptor antagonists Curr Pharm Des 2003 9 707 714 10.2174/1381612033455477 12570788

[B63] Fransioli J Bailey B Gude NA Cottage CT Muraski JA Emmanuel G Wu W Alvarez R Rubio M Ottolenghi S Evolution of the c-kit-positive cell response to pathological challenge in the myocardium Stem Cells 2008 26 1315 1324 10.1634/stemcells.2007-0751 18308948 PMC4037162

[B64] Urbanek K Torella D Sheikh F De Angelis A Nurzynska D Silvestri F Beltrami CA Bussani R Beltrami AP Quaini F Myocardial regeneration by activation of multipotent cardiac stem cells in ischemic heart failure Proceedings of the National Academy of Sciences 2005 102 8692 8697 10.1073/pnas.0500169102 PMC1150816 15932947

[B65] Dawn B Stein AB Urbanek K Rota M Whang B Rastaldo R Torella D Tang XL Rezazadeh A Kajstura J Cardiac stem cells delivered intravascularly traverse the vessel barrier, regenerate infarcted myocardium, and improve cardiac function Proc Natl Acad Sci USA 2005 102 3766 3771 10.1073/pnas.0405957102 15734798 PMC553298

[B66] Raffaghello L Bianchi G Bertolotto M Montecucco F Busca A Dallegri F Ottonello L Pistoia V Human mesenchymal stem cells inhibit neutrophil apoptosis: a model for neutrophil preservation in the bone marrow niche Stem Cells 2008 26 151 162 10.1634/stemcells.2007-0416 17932421

[B67] Mirotsou M Zhang Z Deb A Zhang L Gnecchi M Noiseux N Mu H Pachori A Dzau V Secreted frizzled related protein 2 (Sfrp2) is the key Akt-mesenchymal stem cell-released paracrine factor mediating myocardial survival and repair Proc Natl Acad Sci USA 2007 104 1643 1648 10.1073/pnas.0610024104 17251350 PMC1785280

[B68] Wang M Crisostomo PR Herring C Meldrum KK Meldrum DR Human progenitor cells from bone marrow or adipose tissue produce VEGF, HGF, IGF-I in response to TNF by a p38 MAPK-dependent mechanism Am J Physiol Regul Integr Comp Physiol 2006 291 R880 884 16728464 10.1152/ajpregu.00280.2006

[B69] Urbanek K Rota M Cascapera S Bearzi C Nascimbene A De Angelis A Hosoda T Chimenti S Baker M Limana F Cardiac stem cells possess growth factor-receptor systems that after activation regenerate the infarcted myocardium, improving ventricular function and long-term survival Circ Res 2005 97 663 673 10.1161/01.RES.0000183733.53101.11 16141414

[B70] Li TS Takahashi M Ohshima M Qin SL Kubo M Muramatsu K Hamano K Myocardial repair achieved by the intramyocardial implantation of adult cardiomyocytes in combination with bone marrow cells Cell Transplant 2008 17 695 703 10.3727/096368908786092702 18819257

[B71] Crisostomo PR Wang Y Markel TA Wang M Lahm T Meldrum DR Human mesenchymal stem cells stimulated by TNF-alpha, LPS, or hypoxia produce growth factors by an NF kappa B- but not JNK-dependent mechanism Am J Physiol Cell Physiol 2008 294 C675 682 10.1152/ajpcell.00437.2007 18234850

[B72] Fu X He Y Xie C Liu W Bone marrow mesenchymal stem cell transplantation improves ovarian function and structure in rats with chemotherapy-induced ovarian damage Cytotherapy 2008 10 353 363 10.1080/14653240802035926 18574768

[B73] Zeng F Chen MJ Baldwin DA Gong ZJ Yan JB Qian H Wang J Jiang X Ren ZR Sun D Multiorgan engraftment and differentiation of human cord blood CD34+ Lin- cells in goats assessed by gene expression profiling Proc Natl Acad Sci USA 2006 103 7801 7806 10.1073/pnas.0602646103 16682618 PMC1472525

[B74] Nishiyama N Miyoshi S Hida N Uyama T Okamoto K Ikegami Y Miyado K Segawa K Terai M Sakamoto M The significant cardiomyogenic potential of human umbilical cord blood-derived mesenchymal stem cells in vitro Stem Cells 2007 25 2017 2024 10.1634/stemcells.2006-0662 17495114

[B75] Kawada H Fujita J Kinjo K Matsuzaki Y Tsuma M Miyatake H Muguruma Y Tsuboi K Itabashi Y Ikeda Y Nonhematopoietic mesenchymal stem cells can be mobilized and differentiate into cardiomyocytes after myocardial infarction Blood 2004 104 3581 3587 10.1182/blood-2004-04-1488 15297308

[B76] Vieyra DS Jackson KA Goodell MA Plasticity and tissue regenerative potential of bone marrow-derived cells Stem Cell Rev 2005 1 65 69 10.1385/SCR:1:1:065 17132877

[B77] Fazel S Cimini M Chen L Li S Angoulvant D Fedak P Verma S Weisel RD Keating A Li RK Cardioprotective c-kit+ cells are from the bone marrow and regulate the myocardial balance of angiogenic cytokines J Clin Invest 2006 116 1865 1877 10.1172/JCI27019 16823487 PMC1483161

[B78] Kocher AA Schuster MD Szabolcs MJ Takuma S Burkhoff D Wang J Homma S Edwards NM Itescu S Neovascularization of ischemic myocardium by human bone-marrow-derived angioblasts prevents cardiomyocyte apoptosis, reduces remodeling and improves cardiac function Nat Med 2001 7 430 436 10.1038/86498 11283669

[B79] Strauer BE Brehm M Zeus T Gattermann N Hernandez A Sorg RV Kogler G Wernet P [Intracoronary, human autologous stem cell transplantation for myocardial regeneration following myocardial infarction] Dtsch Med Wochenschr 2001 126 932 938 10.1055/s-2001-16579-1 11523014

[B80] Hamano K Nishida M Hirata K Mikamo A Li TS Harada M Miura T Matsuzaki M Esato K Local implantation of autologous bone marrow cells for therapeutic angiogenesis in patients with ischemic heart disease: clinical trial and preliminary results Jpn Circ J 2001 65 845 847 10.1253/jcj.65.845 11548889

[B81] Abdel-Latif A Bolli R Tleyjeh IM Montori VM Perin EC Hornung CA Zuba-Surma EK Al-Mallah M Dawn B Adult bone marrow-derived cells for cardiac repair: a systematic review and meta-analysis Arch Intern Med 2007 167 989 997 10.1001/archinte.167.10.989 17533201

[B82] Martin-Rendon E Brunskill S Doree C Hyde C Watt S Mathur A Stanworth S Stem cell treatment for acute myocardial infarction Cochrane Database Syst Rev 2008 CD006536 18843721 10.1002/14651858.CD006536.pub2

[B83] Kang S Yang YJ Li CJ Gao RL Effects of intracoronary autologous bone marrow cells on left ventricular function in acute myocardial infarction: a systematic review and meta-analysis for randomized controlled trials Coron Artery Dis 2008 19 327 335 10.1097/MCA.0b013e328300dbd3 18607170

[B84] Zhang SN Sun AJ Ge JB Yao K Huang ZY Wang KQ Zou YZ Intracoronary autologous bone marrow stem cells transfer for patients with acute myocardial infarction: A meta-analysis of randomised controlled trials Int J Cardiol 2009 136 2 178 85 10.1016/j.ijcard.2008.04.071 18644638

[B85] Herrera MB Bussolati B Bruno S Morando L Mauriello-Romanazzi G Sanavio F Stamenkovic I Biancone L Camussi G Exogenous mesenchymal stem cells localize to the kidney by means of CD44 following acute tubular injury Kidney Int 2007 72 430 441 10.1038/sj.ki.5002334 17507906

[B86] Sackstein R Merzaban JS Cain DW Dagia NM Spencer JA Lin CP Wohlgemuth R Ex vivo glycan engineering of CD44 programs human multipotent mesenchymal stromal cell trafficking to bone Nat Med 2008 14 181 187 10.1038/nm1703 18193058

[B87] Zhu H Mitsuhashi N Klein A Barsky LW Weinberg K Barr ML Demetriou A Wu GD The role of the hyaluronan receptor CD44 in mesenchymal stem cell migration in the extracellular matrix Stem Cells 2006 24 928 935 10.1634/stemcells.2005-0186 16306150

[B88] Wang Y Deng Y Zhou GQ SDF-1alpha/CXCR4-mediated migration of systemically transplanted bone marrow stromal cells towards ischemic brain lesion in a rat model Brain Res 2008 1195 104 112 10.1016/j.brainres.2007.11.068 18206136

[B89] Cheng Z Ou L Zhou X Li F Jia X Zhang Y Liu X Li Y Ward CA Melo LG Targeted migration of mesenchymal stem cells modified with CXCR4 gene to infarcted myocardium improves cardiac performance Mol Ther 2008 16 571 579 10.1038/sj.mt.6300374 18253156

[B90] Neschadim A McCart JA Keating A Medin JA A roadmap to safe, efficient, and stable lentivirus-mediated gene therapy with hematopoietic cell transplantation Biol Blood Marrow Transplant 2007 13 1407 1416 10.1016/j.bbmt.2007.09.014 18022569

[B91] Hung SC Pochampally RR Hsu SC Sanchez C Chen SC Spees J Prockop DJ Short-term exposure of multipotent stromal cells to low oxygen increases their expression of CX3CR1 and CXCR4 and their engraftment in vivo PLoS ONE 2007 2 e416 10.1371/journal.pone.0000416 17476338 PMC1855077

[B92] Shi M Li J Liao L Chen B Li B Chen L Jia H Zhao RC Regulation of CXCR4 expression in human mesenchymal stem cells by cytokine treatment: role in homing efficiency in NOD/SCID mice Haematologica 2007 92 897 904 10.3324/haematol.10669 17606439

[B93] Tang YL Qian K Zhang YC Shen L Phillips MI Mobilizing of haematopoietic stem cells to ischemic myocardium by plasmid mediated stromal-cell-derived factor-1alpha (SDF-1alpha) treatment Regul Pept 2005 125 1 8 10.1016/j.regpep.2004.10.014 15582707

[B94] Gibble JW Ness PM Fibrin glue: the perfect operative sealant? Transfusion 1990 30 741 747 10.1046/j.1537-2995.1990.30891020337.x 2219264

[B95] Zhang G Nakamura Y Wang X Hu Q Suggs LJ Zhang J Controlled release of stromal cell-derived factor-1 alpha in situ increases c-kit+ cell homing to the infarcted heart Tissue Eng 2007 13 2063 2071 10.1089/ten.2006.0013 17518719

[B96] Barile L Messina E Giacomello A Marban E Endogenous cardiac stem cells Prog Cardiovasc Dis 2007 50 31 48 10.1016/j.pcad.2007.03.005 17631436

[B97] Tan Y Shao H Eton D Yang Z Alonso-Diaz L Zhang H Schulick A Livingstone AS Yu H Stromal cell-derived factor-1 enhances pro-angiogenic effect of granulocyte-colony stimulating factor Cardiovasc Res 2007 73 823 832 10.1016/j.cardiores.2006.12.015 17258698 PMC2243257

[B98] Latini R Brines M Fiordaliso F Do non-hemopoietic effects of erythropoietin play a beneficial role in heart failure? Heart Fail Rev 2008 13 415 423 10.1007/s10741-008-9084-z 18236153

[B99] Brunner S Winogradow J Huber BC Zaruba MM Fischer R David R Assmann G Herbach N Wanke R Mueller-Hoecker J Erythropoietin administration after myocardial infarction in mice attenuates ischemic cardiomyopathy associated with enhanced homing of bone marrow-derived progenitor cells via the CXCR-4/SDF-1 axis FASEB J 2008 23 2 351 61 10.1096/fj.08-109462 18827024

[B100] Mikirova NA Jackson JA Hunninghake R Kenyon J Chan KW Swindlehurst CA Minev B Patel AN Murphy MP Smith L Circulating endothelial progenitor cells: a new approach to anti-aging medicine? J Transl Med 2009 7 106 10.1186/1479-5876-7-106 20003528 PMC2804590

[B101] Babin-Ebell J Sievers HH Charitos EI Klein HM Jung F Hellberg AK Depping R Sier HA Marxsen J Stoelting S Transmyocardial laser revascularization combined with intramyocardial endothelial progenitor cell transplantation in patients with intractable ischemic heart disease ineligible for conventional revascularization: preliminary results in a highly selected small patient cohort Thorac Cardiovasc Surg 58 11 16 10.1055/s-0029-1186199 20072970

[B102] Chambers SM Shaw CA Gatza C Fisk CJ Donehower LA Goodell MA Aging hematopoietic stem cells decline in function and exhibit epigenetic dysregulation PLoS Biol 2007 5 e201 10.1371/journal.pbio.0050201 17676974 PMC1925137

[B103] Schmidt-Lucke C Rossig L Fichtlscherer S Vasa M Britten M Kamper U Dimmeler S Zeiher AM Reduced number of circulating endothelial progenitor cells predicts future cardiovascular events: proof of concept for the clinical importance of endogenous vascular repair Circulation 2005 111 2981 2987 10.1161/CIRCULATIONAHA.104.504340 15927972

[B104] Wagers AJ Sherwood RI Christensen JL Weissman IL Little evidence for developmental plasticity of adult hematopoietic stem cells Science 2002 297 2256 2259 10.1126/science.1074807 12215650

[B105] Rose RA Jiang H Wang X Helke S Tsoporis JN Gong N Keating SC Parker TG Backx PH Keating A Bone Marrow-Derived Mesenchymal Stromal Cells Express Cardiac-Specific Markers, Retain the Stromal Phenotype and do not Become Functional Cardiomyocytes In Vitro Stem Cells 2008 26 11 2884 92 10.1634/stemcells.2008-0329 18687994

[B106] Makino S Fukuda K Miyoshi S Konishi F Kodama H Pan J Sano M Takahashi T Hori S Abe H Cardiomyocytes can be generated from marrow stromal cells in vitro J Clin Invest 1999 103 697 705 10.1172/JCI5298 10074487 PMC408125

[B107] Tomita S Li RK Weisel RD Mickle DA Kim EJ Sakai T Jia ZQ Autologous transplantation of bone marrow cells improves damaged heart function Circulation 1999 100 II247 256 10567312 10.1161/01.cir.100.suppl_2.ii-247

[B108] De Felice L Tatarelli C Mascolo MG Gregorj C Agostini F Fiorini R Gelmetti V Pascale S Padula F Petrucci MT Histone deacetylase inhibitor valproic acid enhances the cytokine-induced expansion of human hematopoietic stem cells Cancer Res 2005 65 1505 1513 10.1158/0008-5472.CAN-04-3063 15735039

[B109] Bug G Gul H Schwarz K Pfeifer H Kampfmann M Zheng X Beissert T Boehrer S Hoelzer D Ottmann OG Valproic acid stimulates proliferation and self-renewal of hematopoietic stem cells Cancer Res 2005 65 2537 2541 10.1158/0008-5472.CAN-04-3011 15805245

[B110] Lee TM Lin MS Chang NC Inhibition of histone deacetylase on ventricular remodeling in infarcted rats Am J Physiol Heart Circ Physiol 2007 293 H968 977 10.1152/ajpheart.00891.2006 17400721

[B111] Hattori N Imao Y Nishino K Ohgane J Yagi S Tanaka S Shiota K Epigenetic regulation of Nanog gene in embryonic stem and trophoblast stem cells Genes Cells 2007 12 387 396 10.1111/j.1365-2443.2007.01058.x 17352742

[B112] Go MJ Takenaka C Ohgushi H Forced expression of Sox2 or Nanog in human bone marrow derived mesenchymal stem cells maintains their expansion and differentiation capabilities Exp Cell Res 2008 314 1147 1154 10.1016/j.yexcr.2007.11.021 18187129

[B113] Takahashi K Yamanaka S Induction of pluripotent stem cells from mouse embryonic and adult fibroblast cultures by defined factors Cell 2006 126 663 676 10.1016/j.cell.2006.07.024 16904174

[B114] Takahashi K Tanabe K Ohnuki M Narita M Ichisaka T Tomoda K Yamanaka S Induction of pluripotent stem cells from adult human fibroblasts by defined factors Cell 2007 131 861 872 10.1016/j.cell.2007.11.019 18035408

[B115] Hanna J Wernig M Markoulaki S Sun CW Meissner A Cassady JP Beard C Brambrink T Wu LC Townes TM Treatment of sickle cell anemia mouse model with iPS cells generated from autologous skin Science 2007 318 1920 1923 10.1126/science.1152092 18063756

[B116] Kim JB Zaehres H Wu G Gentile L Ko K Sebastiano V Arauzo-Bravo MJ Ruau D Han DW Zenke M Pluripotent stem cells induced from adult neural stem cells by reprogramming with two factors Nature 2008 454 646 650 10.1038/nature07061 18594515

[B117] Okita K Nakagawa M Hyenjong H Ichisaka T Yamanaka S Generation of Mouse Induced Pluripotent Stem Cells Without Viral Vectors Science 2008 322 5903 949 53 10.1126/science.1164270 18845712

[B118] Yang S Lin G Tan YQ Zhou D Deng LY Cheng DH Luo SW Liu TC Zhou XY Sun Z Tumor progression of culture-adapted human embryonic stem cells during long-term culture Genes Chromosomes Cancer 2008 47 665 679 10.1002/gcc.20574 18470900

[B119] Ben-Porath I Thomson MW Carey VJ Ge R Bell GW Regev A Weinberg RA An embryonic stem cell-like gene expression signature in poorly differentiated aggressive human tumors Nat Genet 2008 40 499 507 10.1038/ng.127 18443585 PMC2912221

[B120] Li L Baroja ML Majumdar A Chadwick K Rouleau A Gallacher L Ferber I Lebkowski J Martin T Madrenas J Human embryonic stem cells possess immune-privileged properties Stem Cells 2004 22 448 456 10.1634/stemcells.22-4-448 15277692

[B121] Slavin S Kurkalli BG Karussis D The potential use of adult stem cells for the treatment of multiple sclerosis and other neurodegenerative disorders Clin Neurol Neurosurg 2008 110 9 943 6 10.1016/j.clineuro.2008.01.014 18325660

[B122] von Bonin M Stolzel F Goedecke A Richter K Wuschek N Holig K Platzbecker U Illmer T Schaich M Schetelig J Treatment of refractory acute GVHD with third-party MSC expanded in platelet lysate-containing medium Bone Marrow Transplant 2008 43 3 245 51 10.1038/bmt.2008.316 18820709

[B123] Dunac A Frelin C Popolo-Blondeau M Chatel M Mahagne MH Philip PJ Neurological and functional recovery in human stroke are associated with peripheral blood CD34+ cell mobilization J Neurol 2007 254 327 332 10.1007/s00415-006-0362-1 17345048

[B124] Taguchi A Nakagomi N Matsuyama T Kikuchi-Taura A Yoshikawa H Kasahara Y Hirose H Moriwaki H Nakagomi T Soma T Circulating CD34-positive cells have prognostic value for neurologic function in patients with past cerebral infarction J Cereb Blood Flow Metab 2008 29 1 34 8 10.1038/jcbfm.2008.92 18698330

[B125] Grundmann F Scheid C Braun D Zobel C Reuter H Schwinger RH Muller-Ehmsen J Differential increase of CD34, KDR/CD34, CD133/CD34 and CD117/CD34 positive cells in peripheral blood of patients with acute myocardial infarction Clin Res Cardiol 2007 96 621 627 10.1007/s00392-007-0543-7 17676354

[B126] Herrera MB Bussolati B Bruno S Fonsato V Romanazzi GM Camussi G Mesenchymal stem cells contribute to the renal repair of acute tubular epithelial injury Int J Mol Med 2004 14 1035 1041 15547670

[B127] Seebach C Henrich D Tewksbury R Wilhelm K Marzi I Number and proliferative capacity of human mesenchymal stem cells are modulated positively in multiple trauma patients and negatively in atrophic nonunions Calcif Tissue Int 2007 80 294 300 10.1007/s00223-007-9020-6 17431529

[B128] Ciulla MM Ferrero S Gianelli U Paliotti R Magrini F Braidotti P Direct visualization of neo-vessel formation following peripheral injection of bone marrow derived CD34+ cells in experimental myocardial damage Micron 2007 38 321 322 10.1016/j.micron.2006.08.007 17097882

[B129] Wojakowski W Tendera M Mobilization of bone marrow-derived progenitor cells in acute coronary syndromes Folia Histochem Cytobiol 2005 43 229 232 16382890

[B130] Paczkowska E Larysz B Rzeuski R Karbicka A Jalowinski R Kornacewicz-Jach Z Ratajczak MZ Machalinski B Human hematopoietic stem/progenitor-enriched CD34(+) cells are mobilized into peripheral blood during stress related to ischemic stroke or acute myocardial infarction Eur J Haematol 2005 75 461 467 10.1111/j.1600-0609.2005.00536.x 16313257

[B131] Wang XY Lan Y He WY Zhang L Yao HY Hou CM Tong Y Liu YL Yang G Liu XD Identification of mesenchymal stem cells in aorta-gonad-mesonephros and yolk sac of human embryos Blood 2008 111 2436 2443 10.1182/blood-2007-07-099333 18045971

[B132] Dexter TM Stromal cell associated haemopoiesis J Cell Physiol Suppl 1982 1 87 94 10.1002/jcp.1041130414 7040421

[B133] Bakhshi T Zabriskie RC Bodie S Kidd S Ramin S Paganessi LA Gregory SA Fung HC Christopherson KW Mesenchymal stem cells from the Wharton's jelly of umbilical cord segments provide stromal support for the maintenance of cord blood hematopoietic stem cells during long-term ex vivo culture Transfusion 2008 48 12 2638 44 10.1111/j.1537-2995.2008.01926.x 18798803 PMC3444149

[B134] Huang GP Pan ZJ Jia BB Zheng Q Xie CG Gu JH McNiece IK Wang JF Ex vivo expansion and transplantation of hematopoietic stem/progenitor cells supported by mesenchymal stem cells from human umbilical cord blood Cell Transplant 2007 16 579 585 10.3727/096368907783338181 17912949

[B135] Urban VS Kiss J Kovacs J Gocza E Vas V Monostori E Uher F Mesenchymal stem cells cooperate with bone marrow cells in therapy of diabetes Stem Cells 2008 26 244 253 10.1634/stemcells.2007-0267 17932424

[B136] Ichim TE Solano F Brenes R Glenn E Chang J Chan K Riordan NH Placental mesenchymal and cord blood stem cell therapy for dilated cardiomyopathy Reprod Biomed Online 2008 16 898 905 18549704 10.1016/s1472-6483(10)60159-9

[B137] Murphy MP Wang H Patel AN Kambhampati S Angle N Chan K Marleau AM Pyszniak A Carrier E Ichim TE Allogeneic endometrial regenerative cells: an "Off the shelf solution" for critical limb ischemia? J Transl Med 2008 6 45 10.1186/1479-5876-6-45 18713449 PMC2533293

[B138] Toyoda M Cui C Umezawa A Myogenic transdifferentiation of menstrual blood-derived cells Acta Myol 2007 26 176 178 18646568 PMC2949303

[B139] Cui CH Uyama T Miyado K Terai M Kyo S Kiyono T Umezawa A Menstrual blood-derived cells confer human dystrophin expression in the murine model of Duchenne muscular dystrophy via cell fusion and myogenic transdifferentiation Mol Biol Cell 2007 18 1586 1594 10.1091/mbc.E06-09-0872 17314403 PMC1855042

[B140] Hida N Nishiyama N Miyoshi S Kira S Segawa K Uyama T Mori T Miyado K Ikegami Y Cui C Novel cardiac precursor-like cells from human menstrual blood-derived mesenchymal cells Stem Cells 2008 26 1695 1704 10.1634/stemcells.2007-0826 18420831

[B141] Han X Meng X Yin Z Rogers A Zhong J Rillema P Jackson JA Ichim TE Minev B Carrier E Inhibition of intracranial glioma growth by endometrial regenerative cells Cell Cycle 2009 8 606 610 19197154 10.4161/cc.8.4.7731

[B142] Patel AN Silva F Menstrual blood stromal cells: the potential for regenerative medicine Regen Med 2008 3 443 444 10.2217/17460751.3.4.443 18588464

[B143] Zhong Z Patel AN Ichim TE Riordan NH Wang H Min WP Woods EJ Reid M Mansilla E Marin GH Feasibility investigation of allogeneic endometrial regenerative cells J Transl Med 2009 7 15 10.1186/1479-5876-7-15 19232091 PMC2649897

